# RNAa-mediated epigenetic attenuation of the cell senescence via locus specific induction of endogenous SIRT1

**DOI:** 10.1038/s41598-022-17972-9

**Published:** 2022-09-22

**Authors:** Neda Mokhberian, Kazem Sharifi, Ehsan Soleymaninejadian, Mohamad Eftekhary, Seyed Mahmoud Hashemi, Shohreh Farhadi, Satomi Miwa, Hossein Ghanbarian

**Affiliations:** 1 Department of Medical Biotechnology, School of Advanced Technologies in Medicine, Shahid Beheshti University of Medical Sciences, Tehran, Iran; 2 Cellular and Molecular Biology Research Center, Shahid Beheshti University of Medical Sciences, Tehran, Iran; 3 Department of Biology, Faculty of Science, Shahrekord University, Shahrekord, Iran; 4 Department of Clinical, Surgical, Diagnostics and Pediatric Sciences, University of Pavia, Lombardy, Italy; 5 Department of Immunology, School of Medicine, Shahid Beheshti University of Medical Sciences, Tehran, Iran; 6 Medical Nanotechnology and Tissue Engineering Research Center, Shahid Beheshti University of Medical Science, Tehran, Iran; 7 Biosciences Institute, Edwardson Building, Campus for Ageing and Vitality, Newcastle University, Newcastle upon Tyne, NE4 5PL UK; 8 Urogenital Stem Cell Research Center, Shahid Beheshti University of Medical Sciences, Tehran, Iran

**Keywords:** Molecular biology, Stem cells

## Abstract

SIRT1, a known regulator of cellular senescence, is a therapeutic target for age related disorders and its upregulation is a strategy to improve the cell therapeutic potentials of human mesenchymal stem cell (MSCs). Knockdown of natural antisense transcripts via small activating RNAs (RNAa) is an emerging approach for safe and locus specific gene regulation. We have recently identified a natural antisense transcript at human SIRT1 locus (SIRT1-NAT), the expression of which shows a negative correlation with that of SIRT1. To test the hypothetic upregulation of SIRT1 via knockdown of SIRT1-NAT, in this study we designed a single stranded oligonucleotide (SIRT1-antagoNAT) against the antisense transcript, transfection of which efficiently knocked down the SIRT1-NAT and induced SIRT1 transcription in human MSCs. In addition, activation of SIRT1 transfection via knockdown of SIRT1-NAT in human MSCs enhanced their proliferation and differentiation potentials, reduced senescence associated β-galactosidase activity and reversed the senescence associated molecular alterations. Our findings introduce an RNAa mediated approach for epigenetic induction of endogenous SIRT1 and the consequent attenuation of senescence. Further studies should evaluate the therapeutic potentials of this approach against various age related disorders.

## Introduction

Sirtuin1 (SIRT1), encoded by SIRT1 gene and ubiquitously expressed across various tissues, is well known for its regulatory roles on a wide range of fundamental cell physiological processes^1^. SIRT1 protects the cells against oxidative stress and its dysregulated expression is associated with several diseases^2,3^. Of note, as a positive regulator of cellular senescence and aging^4,5^ SIRT1 has become an attractive protective and/or therapeutic target against various age-related disorders such as neurodegenerative^6^, metabolic^7,8^, and cardiovascular diseases^7,9^. Hence, transcriptional activation of SIRT1 using safe and clinically applicable approaches is of great significance.

Natural antisense transcripts (NATs) are long non-coding RNAs transcribed from the opposite DNA strand of some protein-coding genes, which may regulate gene expression via epigenetic modifications and/or due to complementarity to the corresponding mRNAs^10,11^. Though not ubiquitous among all loci, it has been shown by several research groups including our team that inhibition and/or destruction of NATs via activating RNAs (RNAa) may result in locus specific gene regulation, which appears to be safer, more natural, and more appropriate for clinical use compared with methods of ectopic overexpression using exogenous DNA^12–15^.

We have recently identified an NAT at SIRT1 locus in various human tissues and cell lines^16^ according to the database of in silico prediction of NATs^17^ and its identification in various mouse tissues^18^. As the expression level of SIRT1-NAT showed a negative correlation with that of SIRT1 mRNA^16^, we hypothesized a potential regulatory function between the SIRT1 sense and antisense transcripts. Based on our previous findings^13,15^, gene activation by small RNA could probably operate in the SIRT1 locus, where a natural antisense transcript (NAT) is present. In this study, to investigate a potential regulatory role of antisense transcript in the SIRT1 locus, short single-stranded RNA oligonucleotides (antagoNATs or RNAa)^15^ or siRNA were examined to inhibit the activity of the antisense transcript in human adipose tissue derived mesenchymal stem cells (AD-MSCs). In addition, considering senescence as a major challenge against the most promising source for cell therapy, i.e. human MSCs^19–22^, and according to the known anti-senescence roles of SIRT1^23^, we tested whether small RNA-mediated locus specific induction of SIRT1 could attenuate the senescence in human MSCs. To this end, upon destruction of SIRT1-NAT, the proliferation and differentiation potentials as well as senescence associated molecular alterations were examined in human adipose tissue derived MSCs.

## Material and methods

### Design of modified small oligoribonucleotides targeting SIRT1-NAT

To knockdown SIRT1-NAT, we designed an siRNA and 2 antagoNATs. The 17–19 nucleotide antagoNATs contained phosphorothioate modification plus 2′-O-methyl RNA nucleotides. In addition, control siRNA and antagoNATs were designed. Sequences of the antagoNATs and the siRNA are listed in Supplementary Table 1.

### Cell culture

Isolated MSCs from human adipose tissue^24^ were cultured in Dulbecco’s modified Eagle’s medium (DMDM; Thermo Fisher Scientific, Waltham, MA, USA) containing 10% fetal bovine serum (FBS; Gibco, Grand Island, NY, USA) and 1% penicillin/streptomycin (Beyotime Institute of Biotechnology, Nantong, China) and incubated at 37° C and 5% CO_2_. MSCs were characterized by flow cytometric analysis for the expression of the typical markers CD90, CD73, and CD105 and absence of the hematopoietic markers CD14, CD45, CD34^24^. Every 2–3 days, upon 80% confluency, the cells were subcultured at a ratio of 1:3. MSCs at passages 2 and 7 were used in all experiments.

### Small RNA transfection

5 × 10^4^ MSCs were seeded into each well of 12-well plates. After overnight incubation, the cells were transfected with equal doses (40 pM) of siRNA or antagoNATs using Lipofectamine TM 2000 reagent kit (Invitrogen, Carlsbad, CA, USA) according to the manufacturer’s instructions. The transfection efficiency was similarly and relatively high (~ 80%) for all the oligonucleotides and in different passages of MSCs. 48 h post-transfection, cells were collected for further experiments.

### RNA extraction and real-time qPCR

Total RNA was extracted from the MSCs using Hybrid-R™ RNA Extraction Kit (GeneAll, Seoul, South Korea), followed by treatment with DNase I (RNase-free; Invitrogen, Carlsbad, CA, USA) at 37 °C for 15 min to eliminate DNA contamination. Reverse transcription reactions were performed to synthesize cDNA using RevertAid M-MuLV Reverse Transcriptase Kit (Fermentase, Hanover, Germany) according to the manufacturer’s instructions. RT-qPCR was carried out with the SYBR Green I Master Mix kit (Ampliqon, Copenhagen, Denmark) using the ABI 7500 System (Applied Biosciences, Foster City, CA). The copies of target genes were normalized to reference gene GAPDH RNA level as the internal normalization control. Relative expression was calculated using the 2-ΔΔct method. Sequences of Primers are provided in Supplementary Table 1.

### Population doubling time (PDT) assay

5 × 10^4^ MSCs transfected with SIRT1-antagoNAT at passages 2 and 7 and their relative controls were cultured in DMEM medium supplemented with 10% FBS and underwent serial passages. At the end of each passage, the cells were counted using Neubauer counting chamber. The population doubling time (PDT) was calculated using the formula: PDT = ln2*T/ln (NT/N0), in which T, NT, and N0 stand for culture time, cell number at the end of a passage and cell number at the beginning of a passage, respectively.

### Cell cycle analysis

P2 and P7 MSCs transfected with SIRT1-antagoNAT and their relative controls underwent flow cytometric cell cycle analysis, 48 h post-transfection. After fixation in 70% ethanol, washing in PBS and incubation with RNase A (20 ng/mL;Sigma-Aldrich, Steinheim, Germany) for 30 min at 37 °C, the cells were stained with propidium iodide (1 μg/ml; Sigma-Aldrich, Steinheim, Germany) for 5–10 min. The DNA content was measured as fluorescence intensity detected by fluorescent-activated cell sorter (FACS) (BD FACSCalibur™, BD Biosciences, San Jose, CA, USA) at 488 nm. Cells in different phases of the cell cycle were quantified with Flowjo software (Tree Star, Inc., Ashland, OR).

### Colony formation assay

P2 and P7 MSCs transfected with SIRT1 antagoNAT and their relative control cells were seeded at densities of 100 and 500 viable cells/cm^2^ in 6-well plates. After 14 days of incubation at 37 °C, the cells were fixed with ice-cold methanol for 10 min and stained with 0.5% Giemsa solution (Sigma-Aldrich, Steinheim, Germany). The mean number of colonies was quantified as a measure of colony formation potential.

### Senescence-associated β-galactosidase staining

Senescence-associated β-galactosidase (SA-β-Gal) activity was detected using Senescence Cells Histochemical Staining Kit (Sigma-Aldrich, Steinheim, Germany) according to manufacturer’s instructions. Cells were seeded in a 12-well plate at a density of 2 × 10^4^ per well and incubated at 37 °C with 5% CO_2_ for 2 days. For staining, the cells were washed in PBS, fixed for 5 min at room temperature in the fixation solution and incubated with the staining solution overnight at 37 °C in the absence of CO_2_. The activity of SA-β-Gal was quantified by measuring the percentage of blue-stained cells out of total cells via microscopic observation.

### Western blot analysis

Cell lysates were prepared in the RIPA lysis buffer (Invitrogen, Carlsbad, CA, USA) supplemented with 1 mM protease inhibitor, PMSF (phenylmethylsulfonyl fluoride; Roche, Basel, Switzerland). BCA protein assay kit (Beyotime, Beijing, China) was used to quantify protein contents. Equal amounts of proteins were separated by SDS-PAGE and then immunoblotted onto nitrocellulose membranes. After blocking with 5% skimmed milk in TBS/T (Tris-buffered saline, 0.5% Tween 20), the membranes were incubated with primary antibodies overnight at 4 °C against SIRT1, P53 and GAPDH (1:1000, Santa Cruz Biotechnology, Dallas, Texas, USA) followed by incubation with horseradish peroxidase conjugated anti-mouse (1:5000, Santa Cruz Biotechnology, Dallas, Texas, USA) or anti-rabbit (1:5000, Abcam, Cambridge, MA, USA) secondary antibodies for 1 h at 37 °C. Detection of protein bands was performed using the Electro-Chemi-Luminescence (ECL) kit (Amersham Pharmacia Biotech, Piscataway, NJ). The relative expressions of target proteins were normalized to GAPDH as a reference control.

### Differentiation assays

MSCs underwent osteogenic and adipogenic differentiation as described before^25^. For osteogenesis, MSCs were incubated in low- glucose DMEM supplemented with 10% FBS, 0.1 μM dexamethasone (Sigma-Aldrich, Steinheim, Germany), 10 μM β-glycero-phosphate (Merck, Darmstadt, Hessen, Germany), and 50 μM ascorbate (Sigma-Aldrich, Steinheim, Germany) over a period of 3 weeks, The medium was refreshed twice per week. Osteogenesis was confirmed by detection of extracellular calcium via Alizarin Red S (Sigma-Aldrich, Steinheim, Germany) staining. The cells were fixed and stained with Alizarin Red for 20 min at room temperature. Quantification of osteogenic markers including alkaline phosphatase (ALP), osteocalcin (OCN), and runt‐related transcription factor 2 (Runx2) were performed by qPCR. Quantification of the Alizarin Red S staining was determined using ImageJ particle analysis toolbox.

For adipogenesis, cells were incubated in high-glucose DMEM supplemented with 10% FBS, 250 nM dexamethasone, 0.5 μM 3‐isobutyl‐1‐methylxanthine, 10 μg/ml insulin, and 60 μM indomethacin (all from Sigma-Aldrich) over a 3-week period. Medium refreshment was performed twice a week. Identification of adipocytes was performed by Oil Red O staining. The cells were fixed and stained with 0.5% Oil Red O (Sigma-Aldrich, Steinheim, Germany) in methanol for 20 min at room temperature. Markers of adipogenic lineage including peroxisome proliferator activated receptor α (PPARα), PPARγ, and PPARγ coactivator 1 alpha (PGC1α) were quantified by qPCR. Quantification of the Oil Red O staining was determined using ImageJ particle analysis toolbox.

### Alkaline phosphatase quantification

For quantification of ALP activity, ALP‐LiquiColor assay kit (Merck Millipore, Darmstadt, Germany) was used and the absorbance of released p-nitrophenyl phosphate as the substrate was measured at 405 nm in a microplate reader. Total protein content was determined using BCA protein assay kit and used as a reference for normalization to calculate relative alkaline phosphatase activity.

### Calcium content assay

Cells were homogenized in 0.5 N HCl (Merck, Darmstadt, Germany) for an hour at 25 °C and the extracts were cleared by centrifugation. Calcium Liquicolor kit (Parsazmun, Tehran, Iran) was used to quantify the calcium content of the supernatants, according to the manufacturer’s instructions. The absorbance was read at 570 nm and the total calcium concentration was measured by comparison with serial dilutions of the standard solution.

### Statistical analysis

Statistical analysis was performed using Graphpad Prism 7.03 software (San Diego, Calif). All of the results were expressed as mean ± SD. Differences between two groups were tested by two-tailed Student’s t test. In cases of multiple-group testing, one-way ANOVA in conjugation with Tukey’s test was conducted. The Pearson correlation coefficient was used to analyze the strength of the correlation between the gene expression levels and passage number. *p* values less than 0.05 were considered as statistically significant.

### Ethical approval

All procedures performed in the study were in accordance with the ethical standards of the Iran national committee for ethics in biomedical research and with the 1964 Helsinki declaration and its later amendments or comparable ethical standards. Informed consent was obtained from all the participants. The study was approved by the Bioethics Committee of the Shahid Beheshti University of Medical Sciences (No. IR.SBMU.REC.1397.126).

## Results

### SIRT1-NAT is upregulated in AD-MSCs upon passage related senescence

**Figure 1:**
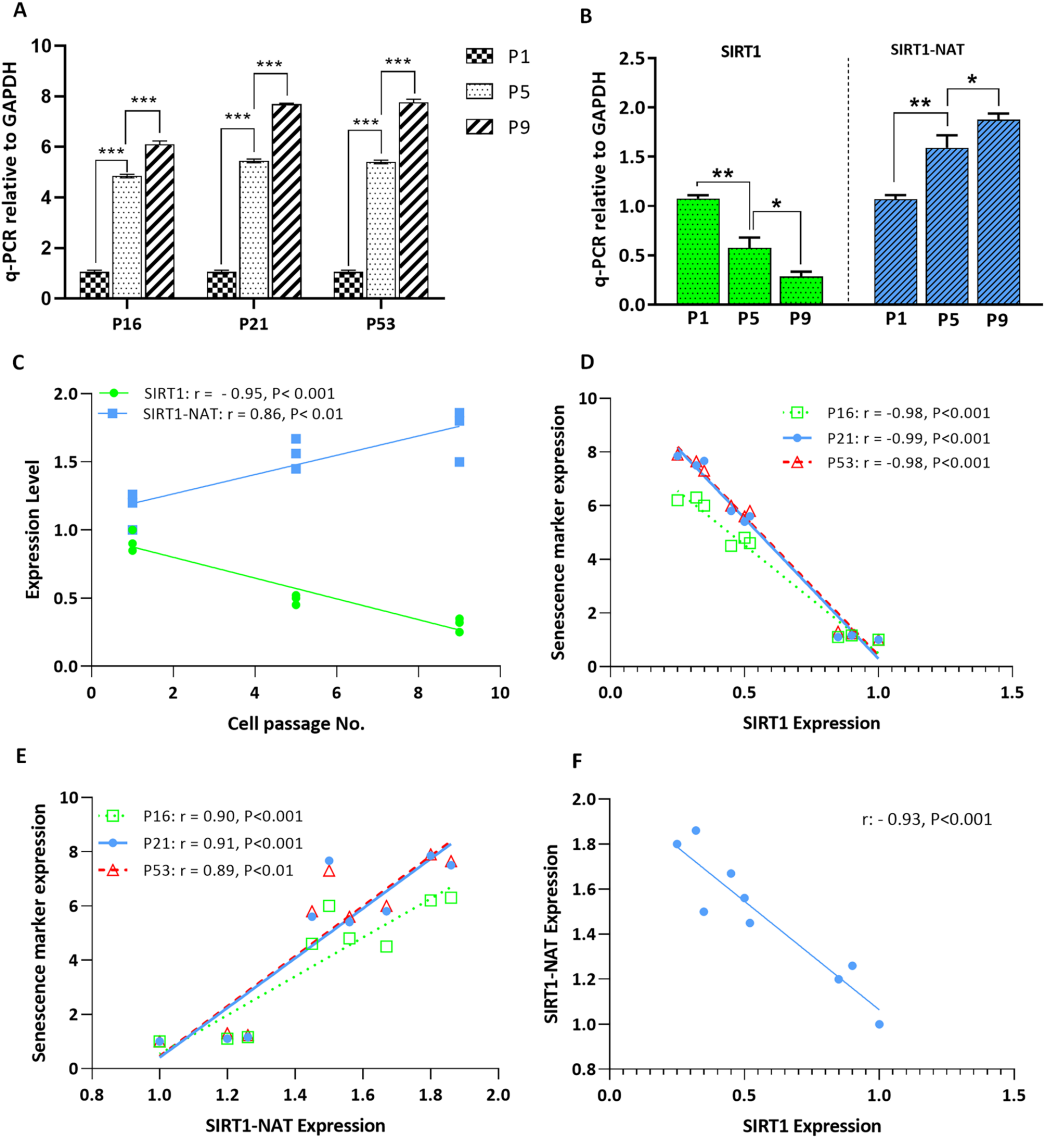
Reverse correlations of SIRT1 and SIRT1-NAT upon increasing passage numbers of MSCs. (**A**) Bar graphs showing the relative expression levels of senescence marker genes P16, P21 and P53 in MSCs at passages 1,5, and 9 determined by RT-qPCR. The expression levels of senescence markers were increased upon subculture of MSCs. (**B**) Bar graphs showing the relative expression levels of SIRT1 mRNA and SIRT1-NAT in MSCs at passages 1, 5 and 9 determined by RT-qPCR. Upon increasing passage number, the expression of SIRT1 was reduced and that of SIRT1-NAT was enhanced. (**C**) Scatter plots showing the negative correlation of SIRT1 and the positive correlation of SIRT1-NAT with passage number of MSCs. (**D**) Scatter plot showing the negative correlation between the expression levels of senescence marker genes and that of SIRT1. (**E**) Scatter plot showing the positive correlation between the expression levels of senescence marker genes and that of SIRT1-NAT. (**F**) Scatter plot showing the negative correlation of SIRT1 and SIRT1-NAT with each other. Data are mean ± SD (n = 3). *p < 0.05, **p < 0.01, ***p < 0.001.

It has been previously reported that SIRT1 is downregulated in hMSCs upon senescence caused by increasing passage number, and once overexpressed, it can reduce senescence in MSCs^23^. During replicative senescence in vitro*,* the population of senescent cells in various cell types including MSCs increases^26–28^. To confirm this in our MSCs, we checked the gene expression levels of P16, P21 and P53 at passages 1, 5 and 9 by quantitative PCR as their increases are known as markers of senescence^27^. The relative expression of P16, P21, and P53 in Passage 5 and Passage 9 MSCs was significantly increased compared with passage 1 and passage 5 MSCs respectively (Fig. 1A). To identify a potential regulatory function of SIRT1-NAT on SIRT1 sense transcript (SIRT1 expression), we first examined the expression level of SIRT1-NAT in hMSCs in association with SIRT1 expression and senescence markers. For this, the expression levels of SIRT1-NAT and SIRT1 were evaluated in P1, P5, and P9 human MSCs by quantitative real time PCR assay. While the expression level of SIRT1 decreased upon increasing passage number of MSCs, the SIRT1-NAT significantly increased in the late passages (Fig. 1B). Correlation analysis confirmed the respectively negative and positive correlations of SIRT1 and SIRT1-NAT with passage number (Fig. 1C). In addition, the expression levels of senescence markers, P16, P21 and P53, showed respectively negative and positive correlations with SIRT1 and SIRT1-NAT (Fig. 1D,E). Consistently, SIRT1 and SIRT1-NAT were negatively correlated with each other (Fig. 1F). These data indicate a potential regulatory function between the SIRT1 sense and antisense transcripts during replicative senescence.

### Small RNA-directed knockdown of SIRT1-NAT activates SIRT1 expression

**Figure 2:**
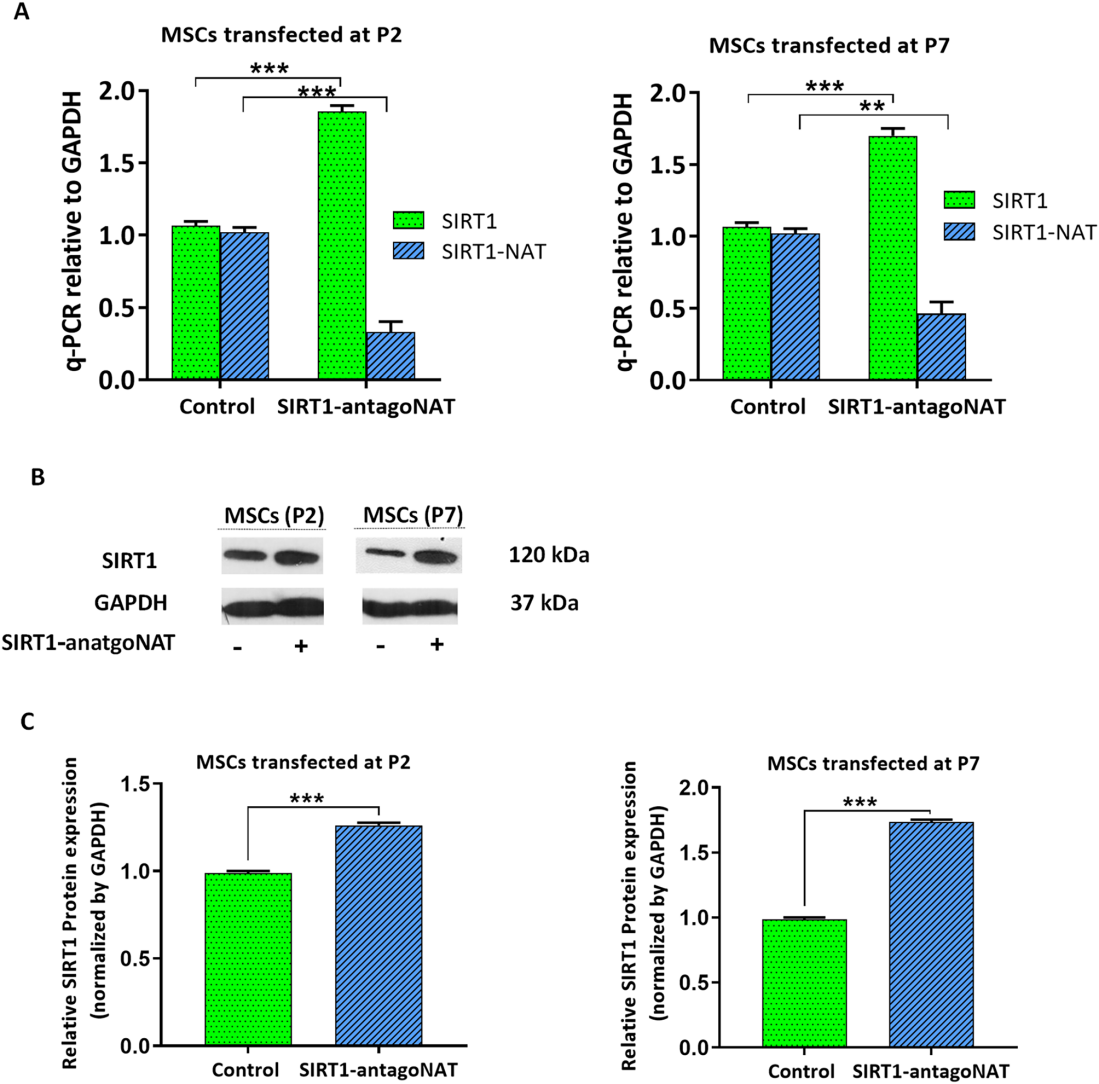
Knockdown of SIRT1-NAT increases SIRT1 mRNA and protein levels in MSCs. (**A**) Bar graphs showing the relative expression of SIRT1 mRNA and SIRT1-NAT in P2 and P7 MSCs transfected with SIRT1-antagoNAT and their controls, determined by RT-qPCR. Treatment with SIRT1-antagoNAT resulted in upregulation of SIRT1 mRNA and downregulation of SIRT1-NAT. (**B**) Representative immunoblots showing the increased expression of SIRT1 protein in P2 and P7 MSCs transfected with SIRT1-antagoNAT compared with controls. Full length blots are presented in Supplementary Fig. 2, (**C**) Bar graphs quantifying the Western blot data represented in B. The expression of SIRT1 protein increased in P2 and P7 MSCs treated with SIRT1-antagoNAT compared with controls. Data are mean ± SD (n = 3). **p < 0.01, ***p < 0.001.

Considering our former^16^ and current reports of negative correlation between the expression levels of SIRT1-NAT and SIRT1 in human cells, we hypothesized that SIRT1 may be upregulated by disruption or inhibition of SIRT1-NAT. To investigate a potential regulatory role of antisense transcript in the SIRT1 locus, short single-stranded RNA oligonucleotides (antagoNATs or RNAa) or siRNA were examined to inhibit the activity of the SIRT1-NAT (Supplementary Fig. 1A, B and Supplementary Table 1). While different small RNAs were examined to inhibit the antisense transcript, one of the antagoNATs could effectively knockdown SIRT1-NAT. Interestingly, knockdown of the antisense transcript resulted in the transcriptional activation of SIRT1, as indicated in Fig. 2A and Supplementary Fig. 1B. The induction of SIRT1 following antagoNAT-mediated destruction of SIRT1-NAT was also confirmed at protein level by Western blotting (Fig. 2B,C).

### Cell proliferation capacity is enhanced by knockdown of SIRT1-NAT

**Figure 3:**
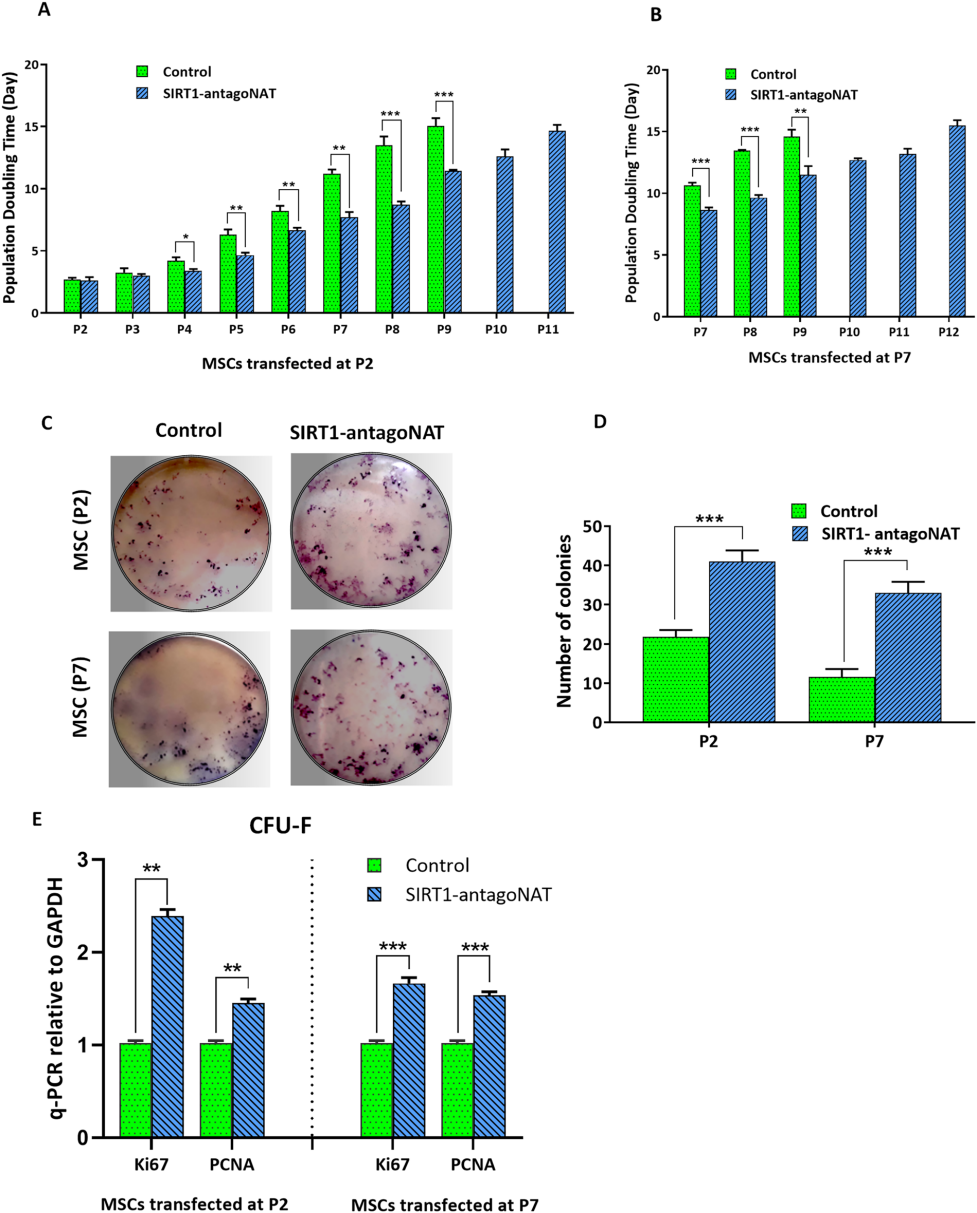
Knockdown of SIRT1-antagoNAT enhances the proliferation of MSCs. (**A**) Bar graphs showing the reduced population doubling time in serial passages of MSCs treated with SIRT1-antagoNAT at P2 compared with controls. (**B**) Bar graphs showing the reduced population doubling time in serial passages of MSCs treated with SIRT1-antagoNAT at P7 compared with controls. In both (**A**,**B**), untreated MSCs could be subcultured no more than passage 10, unlike MSCs treated with SIRT1-antagoNAT. (**C**) Representative photographs of colony formation assay, showing higher number of colonies in P2 and P7 MSCs treated with SIRT1-antagoNAT compared with controls. (**D**) Bar graphs quantifying the results of colony formation assay as represented in (**C**). (**E**) Bar graphs showing the relative expressions of Ki67 and PCNA (proliferation marker genes) in P2 and P7 MSCs treated with SIRT1-antagoNAT and their controls. Data are expressed as mean ± SD (n = 3). *p < 0.05, **p < 0.01, ***p < 0.001.

During senescence, proliferation capacity is known to decline^29^. To examine whether locus specific induction of SIRT1 impacts the proliferation rate in AD-MSCs, P2 and P7 AD-MSCs were transfected with SIRT1-antagoNAT, and we examined doubling time and performed colony forming assays. Transfection of AD-MSCs with SIRT1-antagoNAT at P2 and P7 significantly reduced their doubling time in serial passages and enhanced their maximum passage number (Fig. 3A,B). Moreover, higher number of colonies formed in SIRT1-NAT knockdown P2 and P7 AD-MSCs in comparison with controls (Fig. 3C,D). Furthermore, as evaluated by qPCR, the expression levels of specific proliferation markers, ki67 and PCNA, were higher in SIRT1-antagoNAT treated P2 and P7 MSCs compared with controls (Fig. 3E). In addition, according to the results of flow cytometric cell cycle analysis, the percentage of cells in S phase was higher in SIRT1-antagoNAT transfected P2 and P7 MSCs compared with controls (Supplementary Fig. 3). These data indicate that knockdown of SIRT1-NAT may enable maintenance of the cell proliferation capacity during increases in passage through induction of SIRT1 expression.

### Knockdown of SIRT1-NAT reduces the activity of senescence associated β-galactosidase

**Figure 4:**
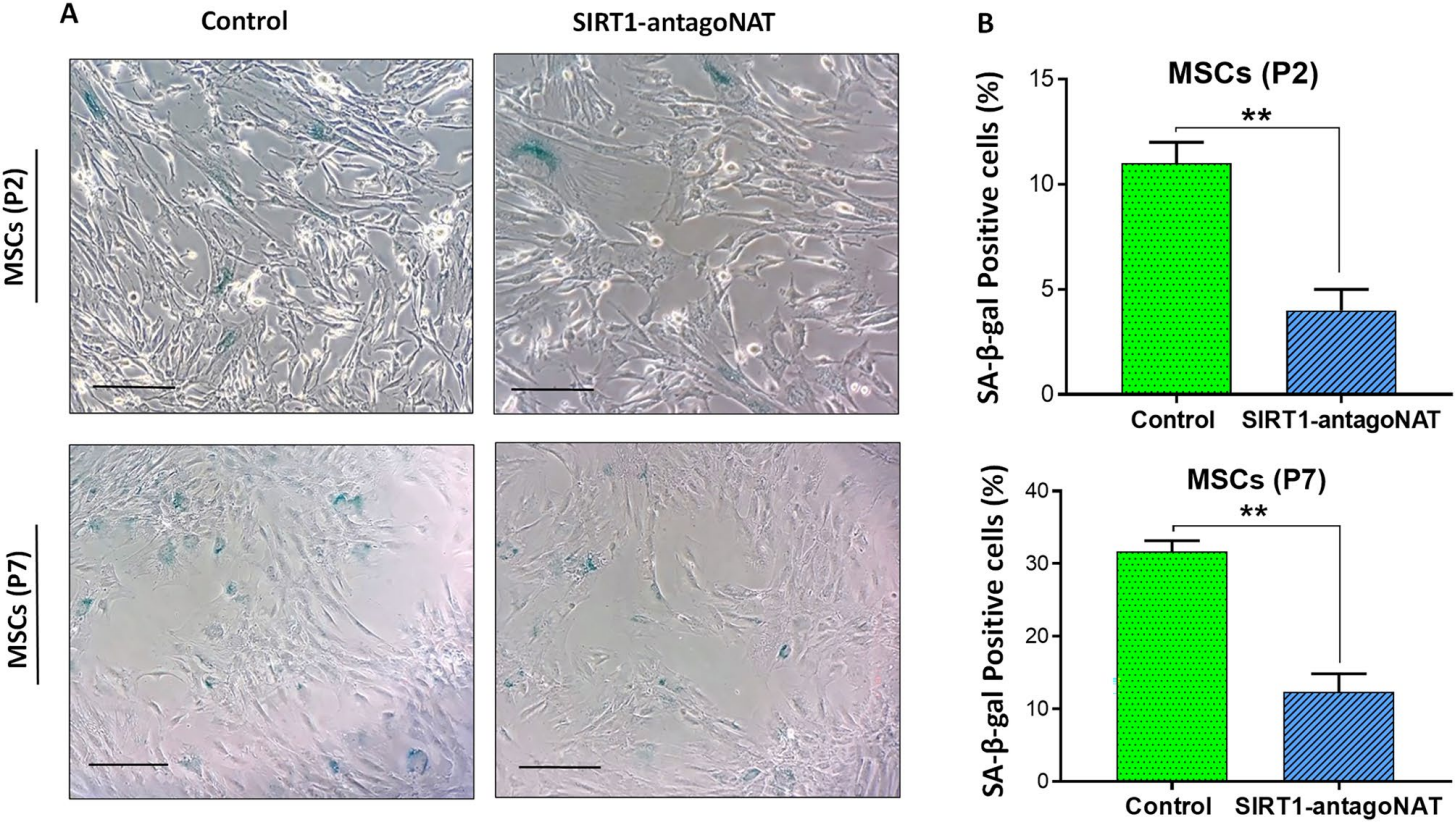
Knockdown of SIRT1-antagoNAT in MSCs reduces the activity of senescence associated β‐galactosidase. (**A**) Representative microscopic images from P2 and P7 MSCs transfected with SIRT1-antagoNAT and their controls being stained for senescence associated β‐galactosidase (SA-β- gal). (**B**) Bar graphs quantifying the results of SA-β gal staining represented in (**A**). A lower percentage of P2 and P7 MSCs transfected with SIRT1-antagoNAT were SA-β-gal + compared with controls. Scale bars: 100 μm. Data are expressed as mean ± SD (n = 3). **p < 0.01.

Anti-senescence roles of SIRT1 have been previously studied^23^. To further examine the role of RNAa-mediated locus specific induction of SIRT1 on senescence, we tested senescence markers in SIRT1-NAT knockdown cells. Senescence associated β-galactosidase (SA-β-gal) activity, which is enhanced in senescent cells^29^, was measured in P2 and P7 MSCs following knockdown of SIRT1-NAT in comparison with controls. Interestingly, a reduced SA-β-gal activity was observed in SIRT1-NAT knockdown cells compared with controls (Fig. 4A,B), further suggesting the anti-senescence role of RNAa- based locus specific induction of SIRT1 via knockdown of antisense transcript in MSCs.

### Senescence associated gene expression characteristics are reversed in MSCs upon knockdown of SIRT1-NAT

**Figure 5:**
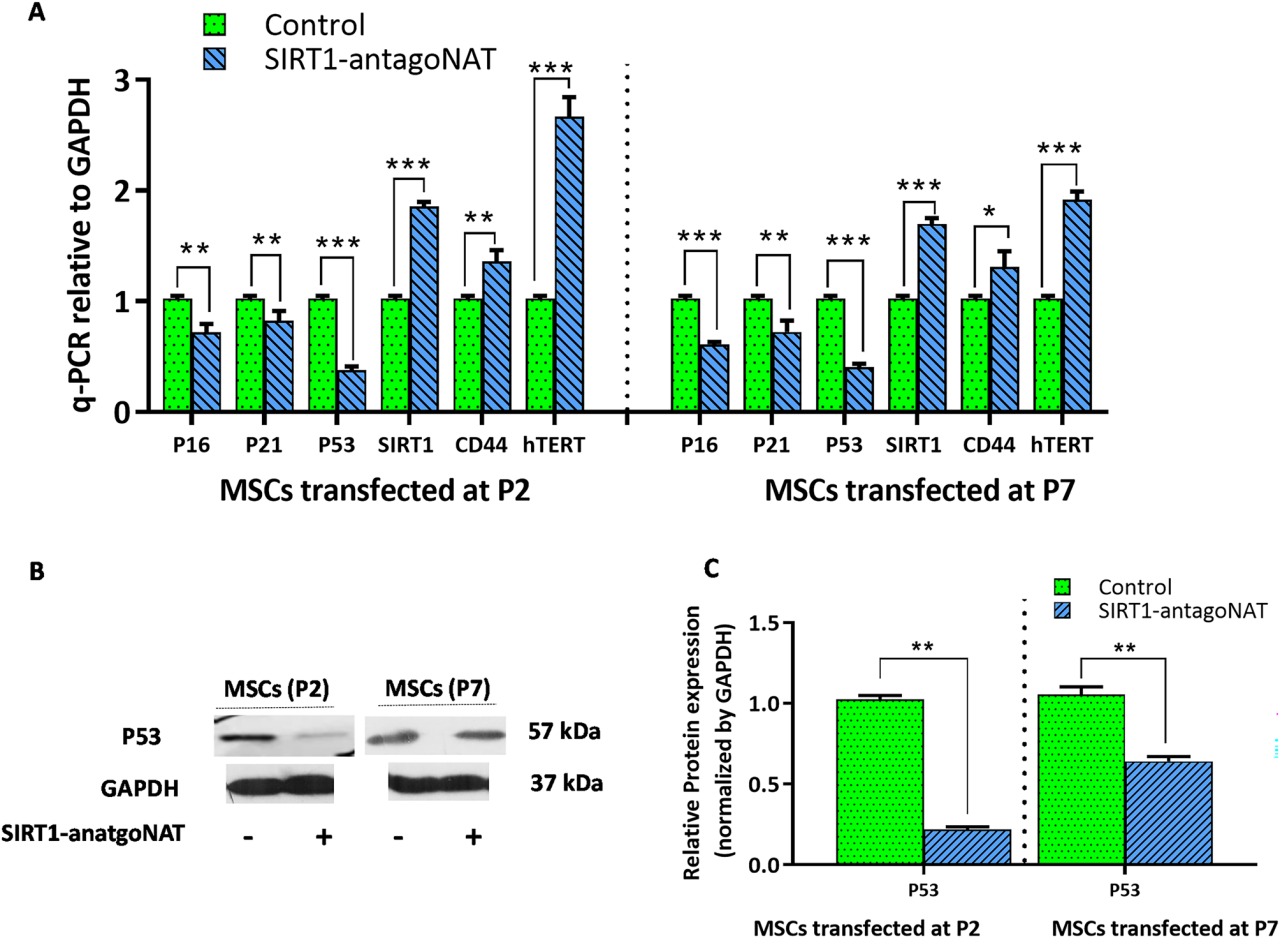
Senescence associated molecular alterations are reversed in MSCs following knockdown of SIRT1-NAT. (**A**) Bar graph showing the relative expressions of P53, P21 and P16 (tumor suppressor genes which characteristically upregulate in senescent cells) as well as that of hTERT and CD44 (which downregulate in senescent MSCs) determined by RT-qPCR. Transfection of SIRT1-antagoNAT in P2 and P7 MSCs downregulated P53, P21 and P16 and upregulated hTERT and CD44 compared with controls. (**B**) Representative immunoblots showing the reduced expression of P53 protein in MSCs transfected with SIRT1-antagoNAT compared with controls. Blots of the GAPDH presented here are the same blots shown in Fig. 2B. Full length blots including P53 bands are presented in supplementary Fig. 2, (**C**) Bar graphs quantifying the Western blot data represented in B. Data are expressed as mean ± SD (n = 3). *p < 0.05, **p < 0.01, ***p < 0.001.

As mentioned above, senescence is also associated with altered expression of several genes. Typically P53, P21, and P16 are upregulated^29^ in senescent cells while such genes as hTERT^30^ and CD44^31^ are downregulated in senescent MSCs. Therefore, the impact of RNAa-mediated induction of SIRT1 on the expression of these genes in P2 and P7 MSCs were assessed by qPCR and Western blot assays. While the expression levels of P53, P21, and P16 significantly reduced in SIRT-NAT knockdown cells, those of hTERT and CD44 enhanced (Fig. 5A). To test such findings at protein levels, the expression of P53 was evaluated by Western blotting, which was significantly decreased in P2 and P7 MSCs transfected with SIRT1-antagoNAT (Fig. 5B,C). These data suggest that RNAa- based locus specific induction of SIRT1 via knockdown of antisense transcript may be able to reverse senescence associated gene expression characteristics in MSCs.

### Senescence associated alterations of differentiation potential is recovered by knockdown of SIRT1-NAT in MSCs

Senescent MSCs show a characteristic general reduction in differentiation potency, in addition to some reports of increased adipogenicity in the expense of reduced osteogenicity^32^. To further support the anti-senescence impacts of RNAa-mediated SIRT1 induction, osteogenic and adipogenic differentiation potentials of MSCs were assessed following knockdown of SIRT1-NAT.

**Figure 6:**
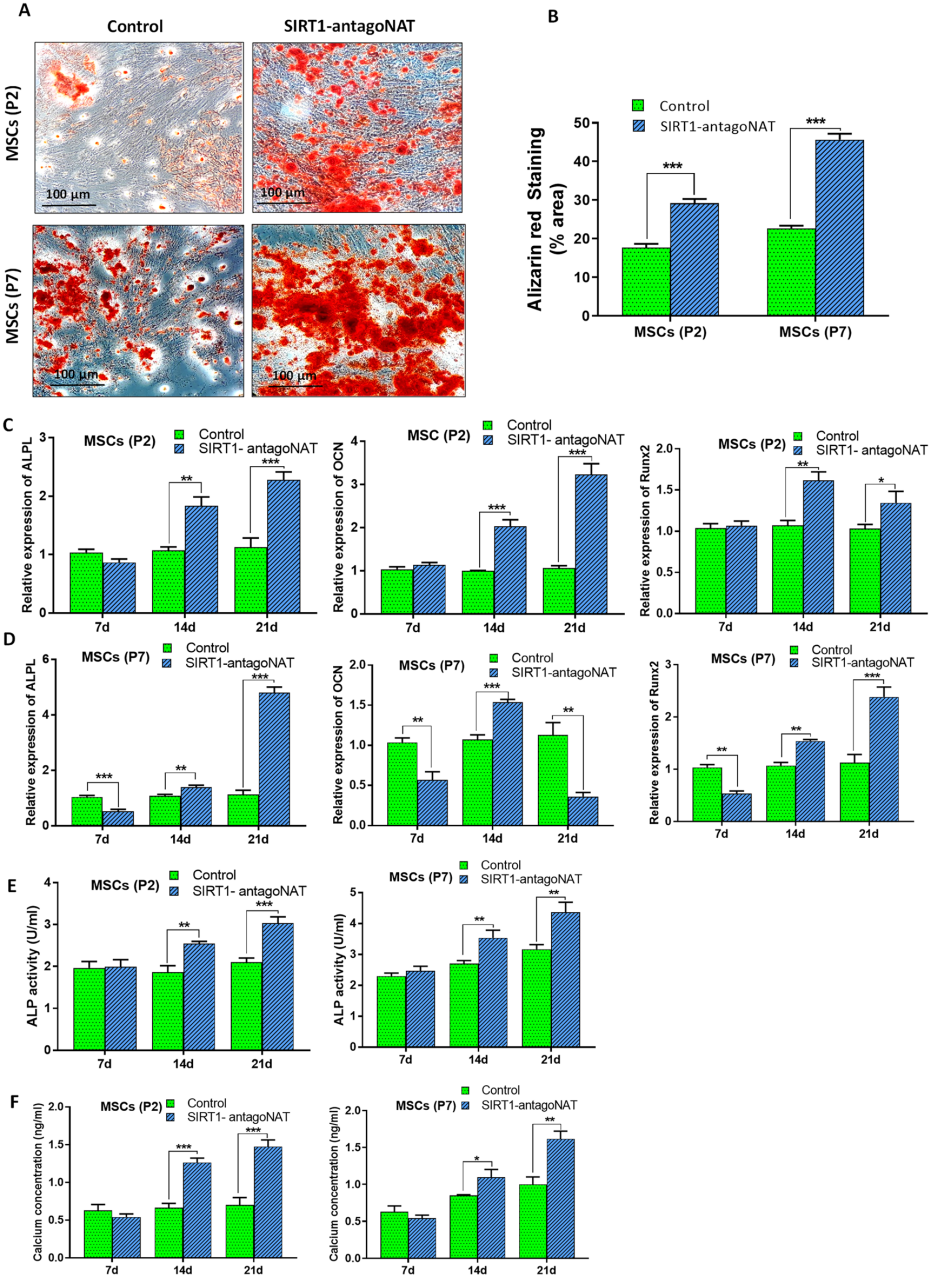
Osteogenic differentiation potentials of MSCs is enhanced following Knockdown of SIRT1-NAT. (**A**) Representative photomicrographs which show the Alizarin red staining following the osteogenic induction of P2 and P7 MSCs transfected with SIRT1-antagoNAT and their controls. (**B**) Bar graphs quantifying the results of Alizarin red staining represented in (**A**). Compared with controls, the percentage of area stained by Alizarin red was higher in SIRT1-antagoNAT treated MSCs directed toward osteogenic fate. (**C**,**D**) Bar graphs showing the relative expressions of ALPL, OCN, and Runx2 (osteoblast marker genes) in P2 (**C**) and P7 (**D**) MSCs treated with SIRT1-antagoNAT and their controls during the 3 weeks period of osteogenic induction, as determined by RT-qPCR. Each time point was used as its own reference. (**E**) Bar graphs showing the higher ALP activity in SIRT1-antagoNAT treated P2 and P7 MSCs during osteogenic induction compared with controls. (**F**) Bar graphs showing the higher calcium content in P2 and P7 MSCs transfected with SIRT1-antagoNAT during the osteogenic induction, compared with controls. Data are expressed as mean ± SD (n = 3). Data in B are obtained from 6 microscopic fields (2 independent experiments). *p < 0.05, **p < 0.01, ***p < 0.001.

Once directed toward osteogenic fate, SIRT1-NAT knockdown cells showed significantly larger area stained by Alizarin red (Fig. 6A,B) and expressed significantly higher levels of osteoblast marker genes including ALPL, OCN, and Runx2 compared with controls as shown by qPCR data (Fig. 6C,D ). In addition, significantly higher levels of ALP activity (Fig. 6E) and calcium content (Fig. 6F) were observed in SIRT1- antagoNAT transfected P2 and P7 MSCs upon osteogenic differentiation. However, expression of OCN downregulated in osteoblasts derived from P7 MSCs at day 21 of osteogenic induction (Fig. 6D).

**Figure 7:**
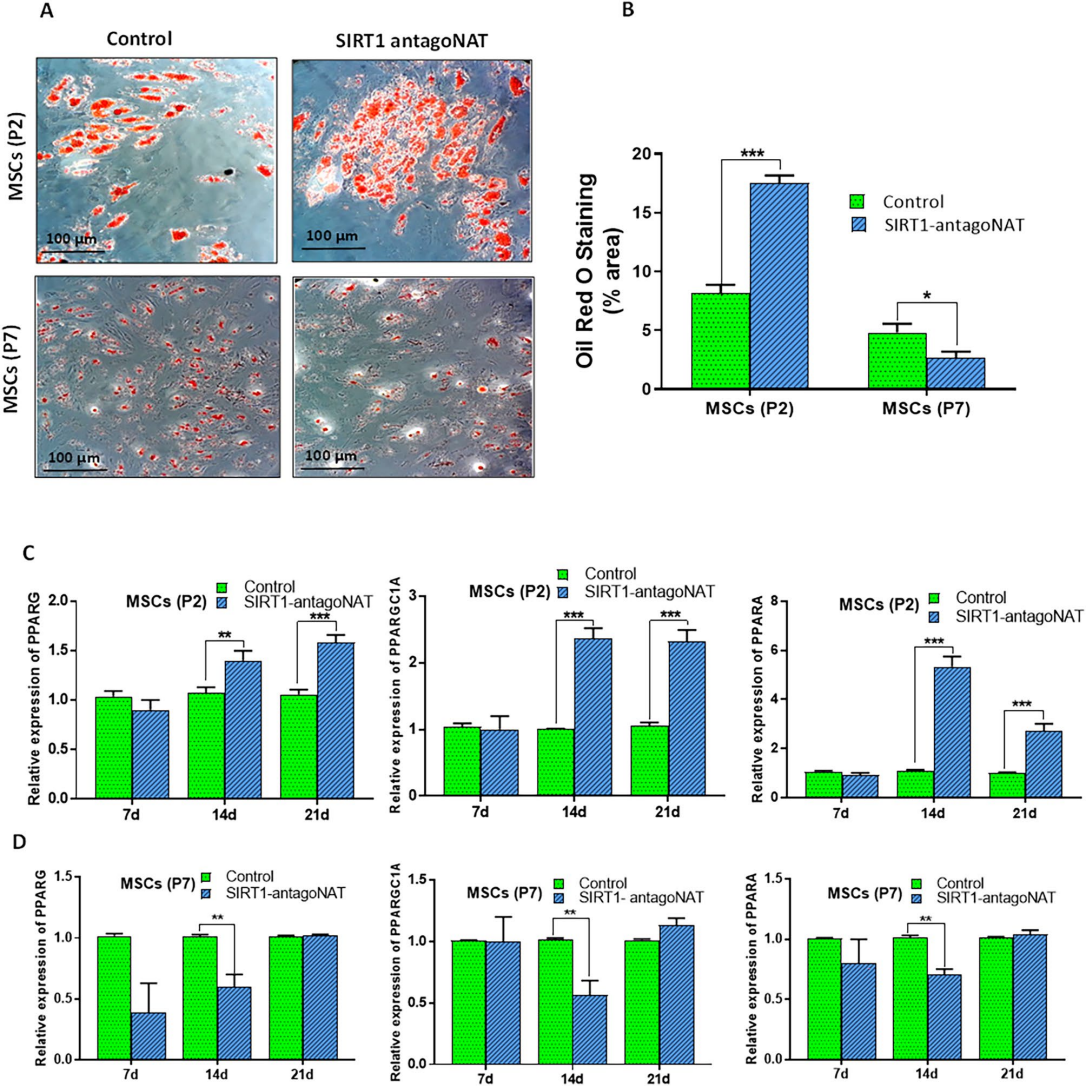
Knockdown of SIRT1-NAT improves the adipogenic differentiation potentials of MSCs. (**A**) Representative photomicrographs which show the Oil Red O staining following the adipogenic induction of P2 and P7 MSCs transfected with SIRT1-antagoNAT and their controls. (**B**) Bar graphs quantifying the results of Oil Red O staining represented in (**A**). Compared with controls, the percentage of area stained by Oil Red was higher in SIRT1-antagoNAT treated P2 MSCs directed toward osteogenic fate. A contrasting result was found in P7. (**C**,**D**) Bar graphs showing the relative expressions of PPARγ, PPARα, and PGC1α (adipogenic marker genes) in P2 (**C**) and P7 (**D**) MSCs treated with SIRT1-antagoNAT and their controls during the 3 weeks period of osteogenic induction, as determined by RT-qPCR. Each time point was used as its own reference. The results of P7 were not consistent with that of P2. Data are expressed as mean ± SD (n = 3). Data in B are obtained from 6 microscopic fields (2 independent experiments). *p < 0.05, **p < 0.01, ***p < 0.001.

In case of adipogenic differentiation, SIRT1-NAT knockdown cells derived from P2 MSCs showed significantly larger area stained by Oil Red (Fig. 7A,B) and expressed significantly higher levels of adipogenic marker genes including PPARγ, PPARα, and PGC1α compared with controls (Fig. 7C,D). The results of adipogenic differentiation upon SIRT1-NAT knockdown in P7 MSCs were different from those of P2 MSCs. The area of Oil Red staining was significantly smaller in SIRT1-NAT knockdown cells derived from P7 MSCs compared with controls (Fig. 7A,B). In addition, significant reductions of adipogenic markers were detected in SIRT1-NAT knockdown P7 MSCs after 14 days of adipogenic induction (Fig. 7D). Altogether, these results suggest that locus specific induction of SIRT1 via knockdown of SIRT1-NAT may have anti-senescence function and may consequently recover the senescence associated alterations of differentiation potency in MSCs.

### Knockdown of SIRT1-NAT induces SIRT1 more efficiently compared with inhibition of miR-34a

**Figure 8:**
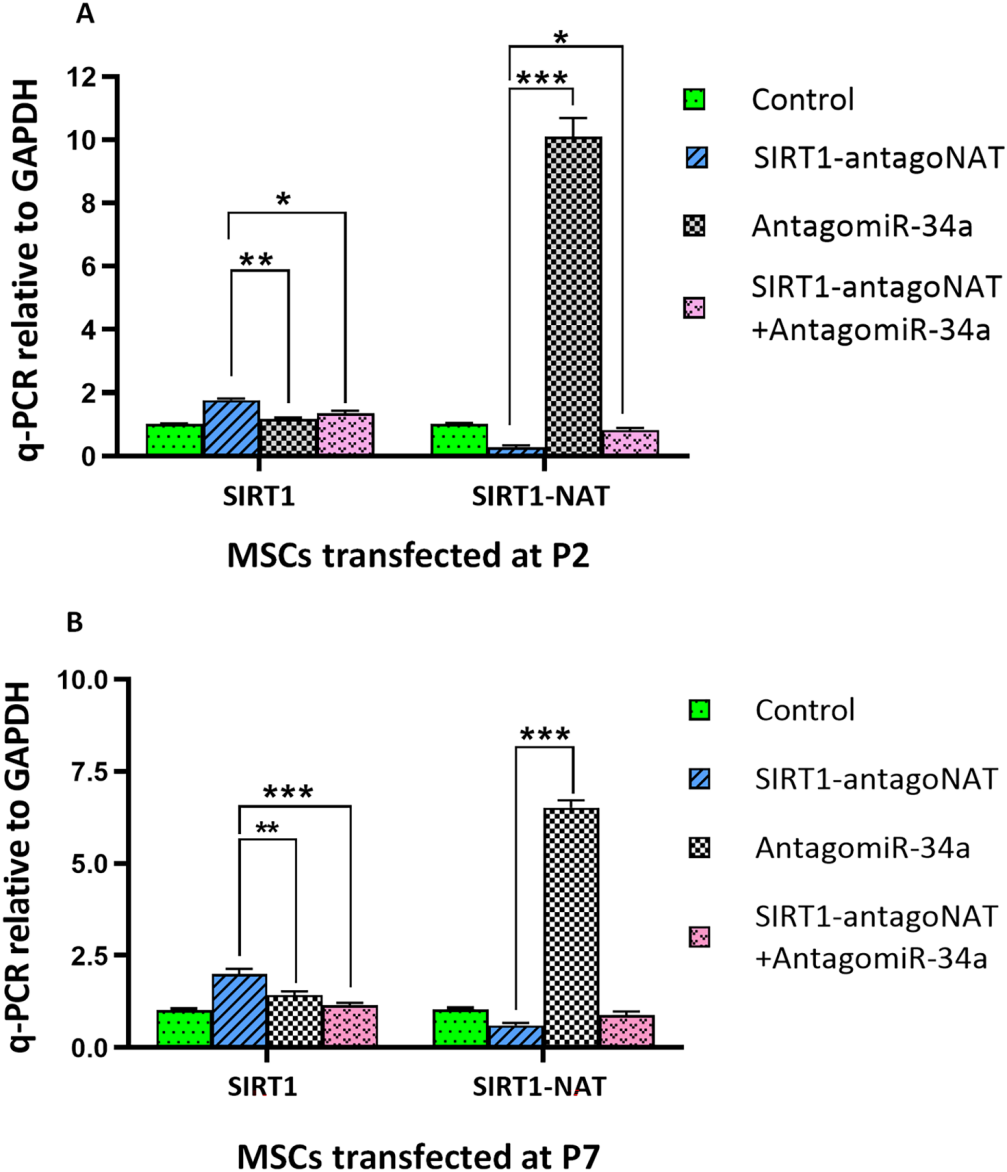
Knockdown of SIRT1-NAT in MSCs more efficiently upregulates SIRT1 compared with inhibition of miR-34a. (**A**,**B**) Bar graphs showing the relative expressions of SIRT1 mRNA and SIRT-NAT determined by RT-qPCR in P2 (**A**) and P7 (**B**) MSCs following treatment with SIRT1-antagoNAT, anti-miR-34a or combination of SIRT1-antagoNAT and anti-miR-34a as well as controls. Treatment with SIRT1-antagoNAT resulted in the highest expression levels of SIRT1 mRNA and the lowest expression levels of SIRT1-NAT. Anti-miR-34a drastically upregulated SIRT1-NAT. Data are expressed as mean ± SD (n = 3). **p < 0.01, ***p < 0.001.

Similar to knockdown of SIRT1-NAT, we have recently reported that inhibition of miR-34a induces SIRT1 expression^33^. To compare the functional consequence of these two approaches in SIRT1 transcriptional activation, SIRT1 was induced following knockdown of SIRT1-NAT and inhibition of miR-34a in MSCs. The cells were transfected with the same dose of SIRT1-antagoNAT and anti-miR-34a. As indicated in Fig. 8, transcriptional activation of SIRT1 induced more efficiently upon SIRT1-NAT knockdown in comparison either with inhibition of miR-34a alone or along with degradation of SIRT1-NAT at P2 and P7 AD-MSCs (Fig. 8A,B). Surprisingly, significant upregulation was observed in the expression of SIRT1-NAT upon inhibition of miR-34a. Moreover, a more efficient differentiation potency was observed following knockdown of SIRT1-NAT compared with that of miR-34a inhibition at both P2 and P7 (Supplementary Fig. 4).

## Discussion

SIRT1 is well known for its protective roles against senescence, aging and age related disorders^3^. Therefore, its upregulation by clinically compatible methods is an attractive therapeutic target. Knockdown of NATs is an emerging approach for locus specific upregulation of target genes with high safety and compatibility to clinical application^14,15^. We have recently shown that small RNA fragments (RNAa) could activate gene expression in a locus specific manner in the absence of exogenous DNA, where a natural antisense transcript is present^13^. Along with our previous studies regarding RNA mediated gene regulation to improve cell based therapeutics, we identified a natural antisense transcript in the SIRT1 locus in human cells and aimed to investigate roles of RNAa-mediated induction of SIRT1 on attenuation of the cell senescence. In this study, we demonstrated that the expression of SIRT1-NAT significantly increased in the late passages while the expression level of SIRT1 decreased in MSCs. This indicated a potential regulatory function between the SIRT1 sense and antisense transcripts during cellular senescence. To investigate a potential regulatory role of antisense transcript in the SIRT1 locus, short RNA molecules (antagoNATs or RNAa) were examined to inhibit the activity of the antisense transcript. Interestingly, antagoNAT-mediated knockdown of SIRT1-NAT resulted in the transcriptional activation of SIRT1. As a proof of the concept, we tested the anti-senescence effect of SIRT1-antagoNAT on MSCs, in which the development of senescent phenotype causes a major problem for clinical use. We found that knockdown of SIRT1-NAT enhances the cell proliferation rate and attenuates the senescence associated decreases in differentiation potency in MSCs. Moreover, we showed that RNAa-directed induction of SIRT1 reduces the SA-β gal activity and reverses the senescence associated gene expression characteristics in MSCs. Altogether, our in vitro results demonstrate that locus specific induction of SIRT1 via antagoNAT–mediated knockdown of SIRT-NAT leads to reprogramming of senescent MSCs to a younger state with improved proliferation and differentiation capacities.

In this study, the anti-senescence effects of SIRT1-antagoNAT were assessed mostly in MSCs from passage 2 and passage 7. It is very likely that in later passages, in which higher percentage of cells are expected to show senescence phenotypes, the anti-senescence effects of SIRT1-antagoNAT may be more evident. Therefore, our hypothesis and current findings should be evaluated under different paradigms of senescence induction, including later passages of MSCs as well as MSCs from young and old individuals.

The transcription of SIRT1 is regulated in a network by several non-coding RNAs. miR-34a is another known regulatory RNA which targets SIRT1 and thereby regulates senescence. In a similar study, we recently showed that anti-miR34a upregulates SIRT1 and reduces senescence in MSCs^34^. Here, we observed significant upregulation in the expression of SIRT1-NAT upon inhibition of miR-34a, indicating a tight regulation of SIRT1 expression and translation in a coordinated manner mediated between at least three RNAs; SIRT1 mRNA, SIRT1-NAT, and miR-34a. In addition to locus specificity as an advantage, our new approach demonstrates a novel regulatory role between SIRT1 sense and antisense transcripts, in which antagoNAT-based knockdown of SIRT1-NAT upregulates SIRT1 more efficiently compared with anti-miR-34a . Furthermore, we observed a more efficient differentiation potency in MSCs upon RNAa-based knockdown of SIRT1 in comparison with miR-34a inhibition, probably because of SIRT1-NAT upregulation following inhibition of miR-34a.

Our current data are clearly consistent with our former ones regarding the negative correlation of SIRT1-NAT and SIRT1 in human cells^16^. However, our findings are in contrast with previous reports of SIRT1-NAT in mouse cells, in which, overexpression of SIRT1-NAT (using plasmid and adenoviral vectors) increased the stability of SIRT1 transcript and possibly promoted its translation and protein level^18,35^. Such contrasts may be explained in part with inter-species differences or differences in methods of induction. We have already shown that transient/ endogenous inductions and continuous/ ectopic inductions may lead to conflicting results^34,36^. Regarding improved differentiation potency of MSCs upon treatment with SIRT1-antagoNAT we obtained some variating data in the late passage. In case of osteogenic differentiation, despite increased expression of osteogenic markers at most of the time points in SIRT1-antagoNAT transfected MSCs, the expression level of all markers at day 7 and that of OCN at day 21 of osteogenesis decreased in passage 7. In case of adipogenic differentiation, treatment of P7 MSCs with SIRT1-antagoNAT reduced their direction toward adipocytes, which is consistent with the former reports of adipogenic shift in senescent MCS^32^. As adipogenicity is not favored in clinical applications of MSCs, making P7 MSCs less adipogenic can be considered an advantage of SIRT-NAT knockdown. We previously found similar variations in our former study using anti-miR-34a to delay senescence in MSCs^34^. Being partly explained by known dysregulations of differentiation potency in aged MSCs^21,32,37^, such variations suggest that anti-senescence strategies should be applied to younger MSCs for higher efficiency.

Our data regarding antagoNAT-mediated induction of SIRT1, further supports the former evidence regarding application of antagoNATs for therapeutic upregulation of genes^12–14^. Without any genetic manipulation, this strategy appears to be safer, more natural and more specific for target gene activation compared with other methods. However, toward clinical application of SIRT1-antagoNAT, several questions remain to be addressed in the upcoming studies including the long term in vivo and in vitro safety and efficiency, in various tissues and organs and in association with various diseases. Comprehensive assessment of anti-senescence effects hypothetically exerted by SIRT1-antagoNAT including DNA damage, autophagy and senescence associated secretory phenotype (SASP) remain to be addressed in future studies. Whether SIRT1-antagoNAT may enhance the enzymatic activity of SIRT1 should be also elucidated. In addition, the exact mechanism(s) by which antagoNAT- mediated knockdown of SIRT-NAT results in transcriptional activation of SIRT1 and protects against senescence are worthy to be investigated. Furthermore, the functionality of MSCs treated with SIRT1-antagoNAT should be further studied both in vitro and in vivo. Notably, unlike normal cells, in cancer cells the anti-senescence effects of SIRT1 is not considered beneficial, as SIRT1 overexpression is observed in several solid cancers and is associated with a poor overall survival^38^. Therefore, in the context of cancer, it can be hypothesized that SIRT1-NAT induction other than disruption might be the appropriate strategy to control the growth of cancer cells.

In conclusion, our data introduce a novel mode of RNA-based regulation in SIRT1 locus for locus specific activation of SIRT1. Our results showing its anti-senescence effects may have therapeutic significance against ageing and age related disorders.

## Supplementary Information

Supplementary Information.
