# Author Correction: RNAa-mediated epigenetic attenuation of the cell senescence via locus specific induction of endogenous SIRT1

**DOI:** 10.1038/s41598-023-28723-9

**Published:** 2023-01-25

**Authors:** Neda Mokhberian, Kazem Sharifi, Ehsan Soleymaninejadian, Mohamad Eftekhary, Seyed Mahmoud Hashemi, Shohreh Farhadi, Satomi Miwa, Hossein Ghanbarian

**Affiliations:** 1 Department of Medical Biotechnology, School of Advanced Technologies in Medicine, Shahid Beheshti University of Medical Sciences, Tehran, Iran; 2 Cellular and Molecular Biology Research Center, Shahid Beheshti University of Medical Sciences, Tehran, Iran; 3 Department of Biology, Faculty of Science, Shahrekord University, Shahrekord, Iran; 4 Department of Clinical, Surgical, Diagnostics and Pediatric Sciences, University of Pavia, Lombardy, Italy; 5 Department of Immunology, School of Medicine, Shahid Beheshti University of Medical Sciences, Tehran, Iran; 6 Medical Nanotechnology and Tissue Engineering Research Center, Shahid Beheshti University of Medical Science, Tehran, Iran; 7 Biosciences Institute, Edwardson Building, Campus for Ageing and Vitality, Newcastle University, Newcastle Upon Tyne, NE4 5PL UK; 8 Urogenital Stem Cell Research Center, Shahid Beheshti University of Medical Sciences, Tehran, Iran

Correction to: *Scientific Reports* 10.1038/s41598-022-17972-9, published online 22 September 2022

The original version of this Article contained an error in the spelling of the author Ehsan Soleymaninejadian which was incorrectly given as Ehsan Soleimaninejadian.

The original Article has been corrected.

