# Supplementary material for: RNAa-mediated epigenetic attenuation of the cell senescence via locus specific induction of endogenous SIRT1
Source: Sci Rep. 2022 Sep 22;12:15826. doi: 10.1038/s41598-022-17972-9 (PMC9500079; doi:10.1038/s41598-022-17972-9)
Supplement: Supplementary file 1 — Supplementary Information. [file 41598_2022_17972_MOESM1_ESM.docx]

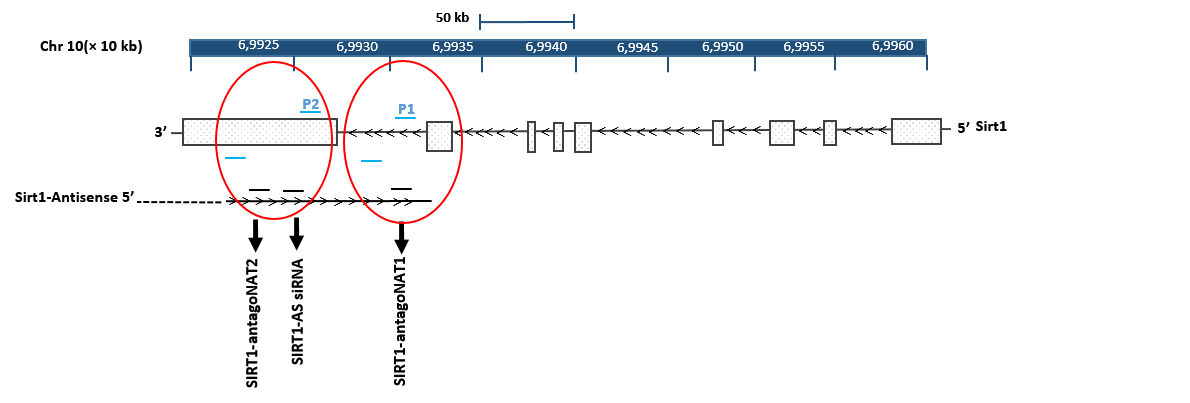


**Supplementary Figure 1**

(**A**) AntagoNATs and siRNA complementary to the SIRT1-NAT sequence, targeting different regions of SIRT1-NAT transcript, were designed to knockdown SIRT1-NAT.

(**B**) The expression levels of SIRT1 mRNA and SIRT1-NAT were determined by quantitative real-time PCR 48 hours after transfection with oligonucleotides. Upregulation of SIRT1 mRNA was observed following treatment of cells with SIRT1-antagoNAT2.

**Supplementary Figure 2**


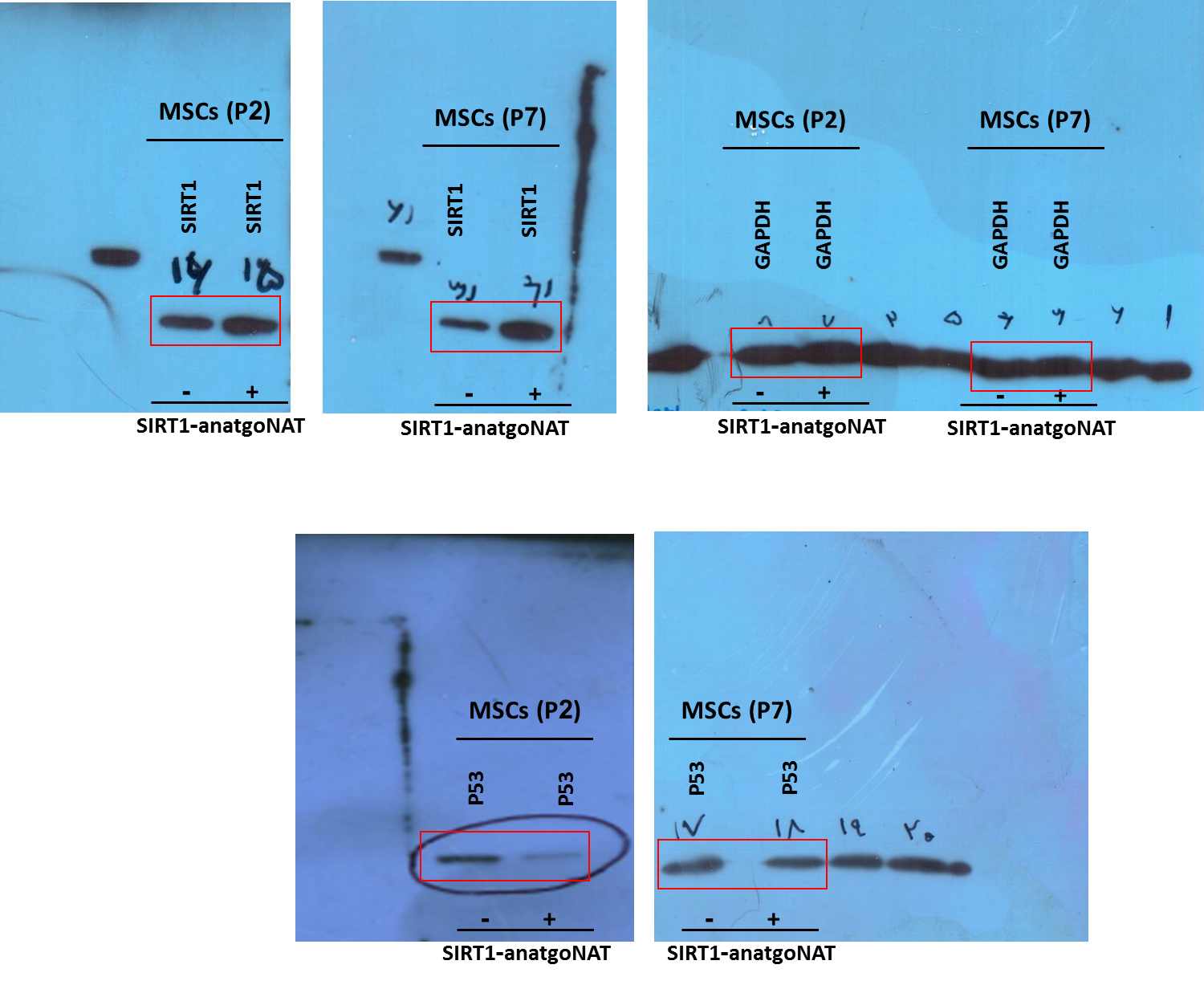


Full length western blot images for figure 2B and figure 5B.

**Supplementary Figure 3**


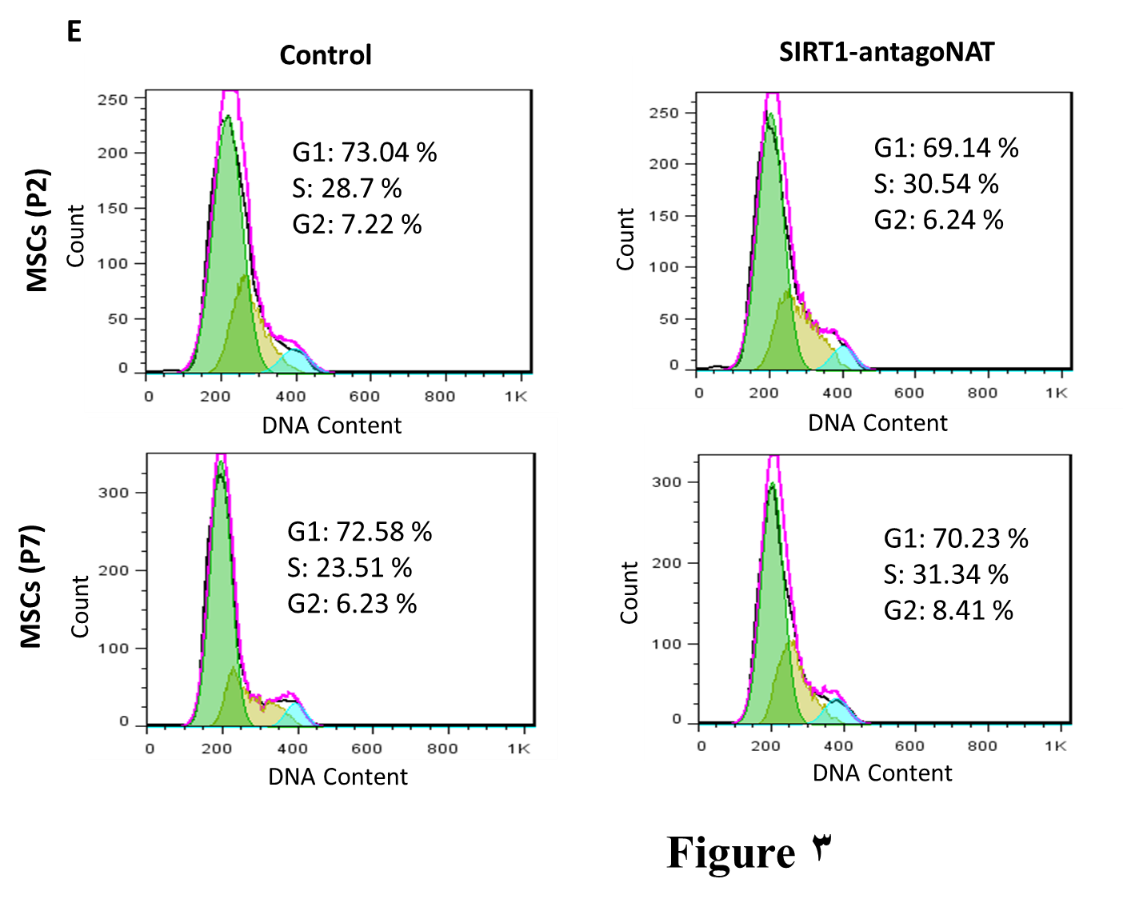


Flow cytometric cell cycle analysis of SIRT1-antagoNAT transfected P2 and P7 MSCs and control MSCs.

**Supplementary Figure 4**

**Adipogenic differentiation (Oil Red O staining)**


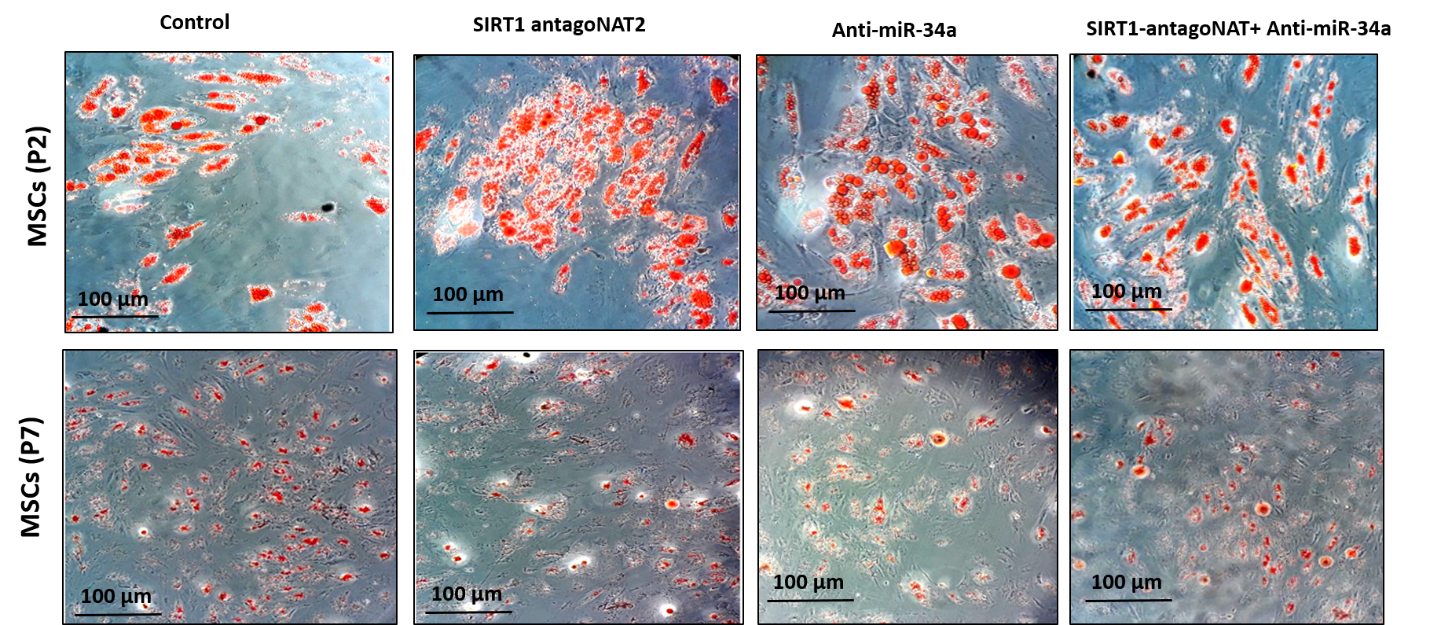
**(A)**

(B)

(**A**) Representative photomicrographs showing the Oil Red O staining following the adipogenic induction of P2 and P7 MSCs transfected with SIRT1-antagoNAT, anti-miR-34a, SIRT1-antagoNAT and anti-miR-34a, and controls. (**B**) Bar graphs quantifying the results of Oil Red O staining represented in A.

**Osteogenic differentiation (Alizarin Red staining)**


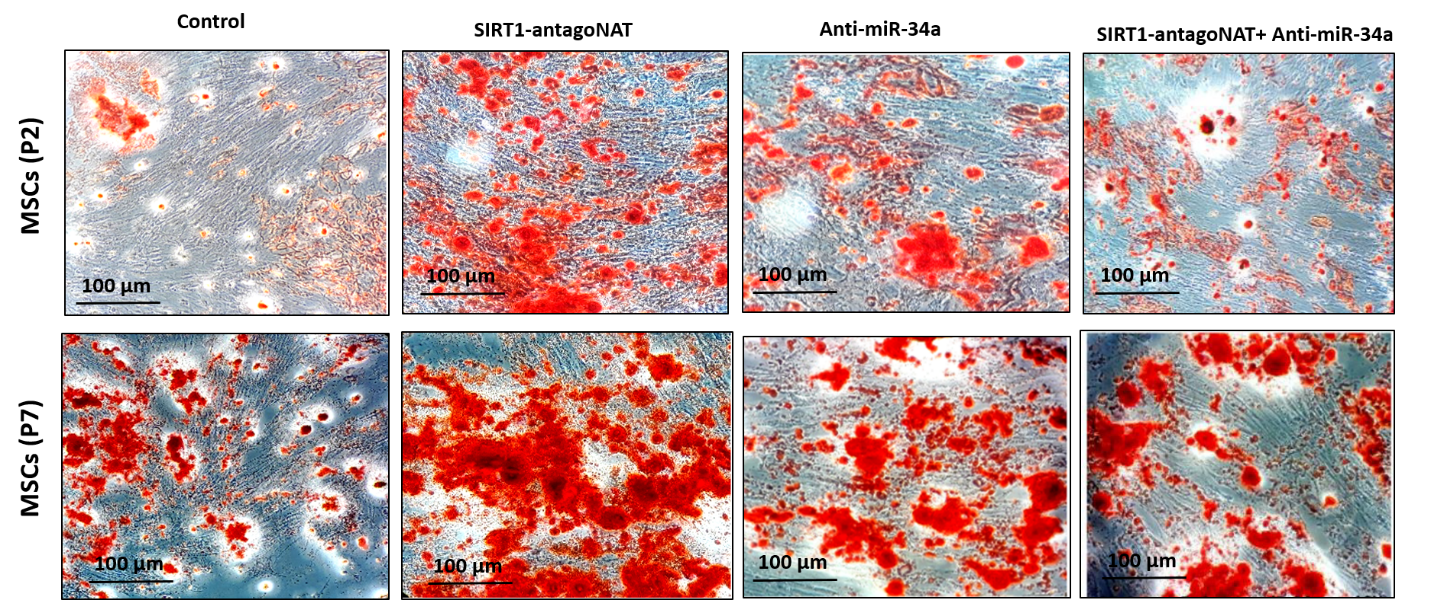
(C)

(D)

(C) Representative photomicrographs showing the Alizarin red staining following the osteogenic induction of P2 and P7 MSCs transfected with SIRT1-antagoNAT, anti-miR-34a, SIRT1-antagoNAT and anti-miR-34a, and controls. (D) Bar graphs quantifying the results of Alizarin red staining represented in C.

**Supplementary Table 1: Sequence information**

| **Application** | **Sequence** | | **Primer name** |
| --- | --- | --- | --- |
| Real-time PCR | | F: GCAAAGGAGCAGATTAGTAGGC  R: GTTACTGCCACAAGAACTAGAGG | SIRT1 |
| Real-time PCR | | F: AGGCACAGAGGTACACTTAC  R: ATACAAGCAATACACTTAGTCCTG | SIRT1-NAT-1 |
| Real time PCR | | F: ATTGGCTCATAAAACTAACCTG  R: GCATATTCACATTTTGGCAGT | SIRT1-NAT-2 |
| Real-time PCR | | F: CAACAGTCGAAGAAGGTGTG  R: TGGTCTGGAGTTTCTGACG | CD44 |
| Real-time PCR | | F: TTCCTCAGCTATGCCCGGACCT  R: TGACACTTCAGCCGCAAGACCC | hTERT |
| Real-time PCR | | F: CATCACGGACACGCTTTC  R: CGGTCGCACTTGTCATAC | PPARα |
| Real-time PCR | | F: TGCGGGATGATGGAGAC  R: GTGGAAGCAGGGTCAAAG | PGC1α |
| Real-time PCR | | F: TGGATCTCTCCGTAATGG  R: GCACTTTGGTACTCTTGAAG | PPARγ |
| Real-time PCR | | F: GCCTTCAAGGTGGTAGCCC  R: CGTTACCCGCCATGACAGTA | RUNX2 |
| Real-time PCR | | F: GCACCTGCCTTACTAACTC  R: AGACACCCATCCCATCTC | ALPL |
| Real-time PCR | | F: CAAAGGTGCAGCCTTTGTGTC  R: TCACAGTCCGGATTGAGCTCA | OCN |
| Real-time PCR | | F: AGCTGCTGCTGCTCCACGG  R: ACCAGCGTGTCCAGGAAGCC | P16 |
| Real-time PCR | | F: CTGGCTAGACGAAGACTCAG  R: AGGCAGTGCTCGCTTGGTAC | P53 |
|  | | F: AAAATACGTGAACAGGAGCCA  R: GAGTCATCTGCGGTACTGTC | Ki67 |
|  | | F: CGCCCTGGTTCTGGAGGTAA  R: CCGGCTGAGACTTGCGTA | PCNA |
| Real-time PCR | | F: TCTGACTTCAACAGCGACACC  R: GTTGCTGTAGCCAAATTCGTT | GAPDH |
| Blocking | | CAAAGAUACAAUAAAUGGC CAAAGAUACAAUAAAUGGC | SIRT1-NAT siRNA |
| Blocking | | AAAUACCUAUCACUGUGGU | SIRT1-antagoNAT1 |
| Blocking | | AGAAACATTAGTGCCUG | SIRT1-antagoNAT2 |
